# Dietary Inclusion Effects of Dried Mealworm, Hydrolyzed Mealworm, Fermented Poultry By-Product, and Hydrolyzed Fish Soluble Protein on Weaning Pigs’ Performance, Fecal Score, and Blood Profiles

**DOI:** 10.3390/ani15111507

**Published:** 2025-05-22

**Authors:** Usman Kayode Kolawole, Kye Jin Lee, In Ho Kim

**Affiliations:** 1 Department of Animal Biotechnology, Dankook University, Cheonan 31116, Republic of Korea; kolawoleusman@rocketmail.com (U.K.K.); jookyjin1234@naver.com (K.J.L.); 2 Smart Animal Bio Institute, Dankook University, Cheonan 31116, Republic of Korea

**Keywords:** poultry by-product, blood profiles, piglets

## Abstract

This study was conducted to investigate the effects of the addition of dried mealworm, hydrolyzed mealworm, fermented poultry by-product meal (FPBM), and hydrolyzed fish soluble protein (HFSP) on the growth performance, nutrient digestibility, fecal score, and blood profiles of weaning pigs. A total of 40 (21-day-old) weaned piglets ([Yorkshire × Landrace] × Duroc) with an initial average body weight (BW) of 7.14 ± 1.29 kg were randomly assigned to one of four treatments for 35 days. There were four treatment groups with five replicates and two pigs (male and female) per pen. The dietary treatment included TRT1, a basal diet + 10% dried mealworm; TRT2, a basal diet + 10% hydrolyzed mealworm; TRT3, a basal diet + 10% fermented poultry by-product meal (FPBM); and TRT4, a basal diet + 10% hydrolyzed fish soluble protein (HFSP). The inclusion of FPBM in weaning pig diets had a significant effect on the blood profile with blood urea nitrogen (BUN) levels (*p* < 0.05), but not on other blood profiles of serum IGF-1, IgG, and IgA concentrations. BUN concentration decreased in response to the dietary inclusion of FPBM (*p* = 0.019). BUN concentration decreased and was at its lowest with FPBM (6.3 mg/dL), followed by hydrolyzed mealworm (7.3 mg/dL), while increasing with dried mealworm and HFSP (8.5 mg/dL). There was no significant difference in the growth performance, nutrient digestibility, and fecal score of piglets fed a basal diet with dried mealworm, hydrolyzed mealworm, FPBM, and HFSP. Hence, the addition of fermented poultry by-products in weaning pigs’ diets decreases the blood urea nitrogen, without any detrimental effect on performance, nutrient digestibility, or fecal score.

## Simple Summary

Inclusion of different sources of protein into the diets of weaning pigs can affect their performance; however, the precise effects vary depending on the pig’s age and the type of protein utilized. In addition to the widely used plant-based protein sources like soybean meal, animal-based sources, including animal products, and fish meal can also be employed. This study examined the effect of the addition of dried mealworm, hydrolyzed mealworm, fermented poultry by-product meal (FPBM), and hydrolyzed fish soluble protein (HFSP) on the growth performance, nutrient digestibility, fecal score, and blood profiles of weaning pigs. The study findings confirmed that there was no major or detrimental effect on the growth performance, nutrient digestibility, fecal score, or blood parameters of weaning pigs, but that BUN concentration decreased in response to dietary inclusion of FPBM.

## 1. Introduction

Various protein sources, both plant- and animal-based, such as soybean, cottonseed meal, meat and bone meal, and fish meal, impact the health and performance of piglets [1]. Among these, soybean is the most commonly used protein source in pig feed due to its excellent amino acid composition and high digestibility [2]. However, its production is linked to several challenges, including environmental production and transportation costs as well as feed/food competition [2]. To enhance feed consumption in newly weaned pigs, highly palatable and nutrition-dense protein sources, such as fish meal, are frequently added to weaning pig diets. It is unknown whether the amount of fish soluble in fish meal influences pig growth performance [3].

Aside from the standard proteins used in animal feed, various alternative protein sources are becoming increasingly popular around the world [4]. In recent years, there has been increased interest in the utilization of non-traditional/non-conventional animal protein sources as alternate feed options for animal production. Alternative animal protein sources, including insects, have the potential to substantially lower feed expenses, which make up 80% of total production costs [5]. Additionally, animal by-products such as dung, offal, viscera, feathers, fish silage, bone, and blood can also serve as cost-effective protein sources [6]. Non-conventional protein feeds’ low nutritional value, excessive unpredictability, and poor palatability have limited their use in swine production. Fermentation technology is the key to overcoming these problems [7]. Pig diets supplemented with poultry by-products (animal protein) demonstrated improvements in apparent digestibility and serum parameters—all of which are powerful markers of an animal’s overall health [8].

Poultry by-product meal (PBM) is a viable protein source for pigs due to its availability and quality [9]. Pig productivity, digestion, and gut health can all be enhanced by fermenting poultry byproducts for use as pig feed [7]. Despite the numerous obstacles that fermentation of non-traditional protein feeds must overcome, there are still a number of encouraging patterns that merit further investigation [7].

However, as conventional protein sources have been commonly used, the use of alternative protein is still contracting [10]. From the above study, we hypothesized that dietary addition of fermented poultry by-product meal (FPBM) improves blood profile in weaning pigs over dried mealworm, hydrolyzed mealworm, hydrolyzed fish soluble protein (HFSP). Therefore, this study aimed to observe the dietary effects of dried mealworm, hydrolyzed mealworm, fermented poultry by-product meal (FPBM), and hydrolyzed fish soluble protein (HFSP) on the growth performance, nutrient digestibility, fecal score, and blood profiles of weaning pigs.

## 2. Materials and Methods

All procedures used in the experiment were revised and accepted by the Institutional Animal Care and Use Committee of Dankook University, Republic of Korea (Ethical approval No. DK-1-1929).

### 2.1. Sources of Experimental Diets Used

Dried mealworm was obtained from Daehan Feed Company Limited (Incheon, Republic of Korea). Hydrolyzed mealworm (HML) (*Tenebrio molitor*) protein powder and hydrolyzed fish soluble protein (HFSP) powder were purchased from KEIL. Poultry by-products, which are easily obtainable as agricultural waste, were obtained from poultry slaughterhouses. The poultry by-products obtained were ground with a meat grinder and then mixed with 4% dextrose. The product was mixed, then passed through a flowtherm heat exchanger, and pasteurized for four minutes at a core temperature of 90 °C. Following heating, the material was allowed to cool to around 20 °C before the addition of a starter culture (10^6^ colony-forming units per gram of Lactobacillus plantarum, a probiotic bacterium known to enhance anti-infective properties against pathogens). The pH level was kept at 4.0. For at least 21 days, fermentation and storage were conducted at 15 °C in 15 L screw-capped barrels following the guidelines of Yoo [11].

### 2.2. Animal Husbandry, Diet, and Experimental Design

A total of 40 weaned piglets ([Yorkshire × Landrace] × Duroc), 21 days old, with an initial average body weight (BW) of 7.14 ± 1.29 kg, were randomly assigned to one of four treatments for 35 days. The pigs were randomly assigned to one of four nutritional treatments according to their BW and gender (male and female). Each treatment comprised five replicates with a male and female per pen. The dietary treatment included TRT1, basal diet + 10% dried mealworm; TRT2, basal diet + 10% hydrolyzed mealworm; TRT3, basal diet + 10% fermented poultry by-product meal (FPBM); and TRT4, basal diet + 10% hydrolyzed fish soluble protein (HFSP). The trial period was divided into 2 phases: phase 1, days 0–15, and phase 2, days 16–35. The NRC [12]) criteria for diet formulation for weaning pigs were adhered to in our trial (Table 1, Table 2 and Table 3). Each piglet occupied a 0.26 m × 0.53 m space in a room that was kept up properly and equipped with a mechanical aeration mechanism. During the experiment, the piglets had unrestricted access to water and food (ad libitum) from pens equipped with a feeder and a nipple drinker. There was artificial light available twelve hours a day. The room’s ambient temperature was maintained at 30 °C for the first week before being reduced by 1 °C each week following that.

### 2.3. Sample Collection

#### 2.3.1. Growth Performance

The individual body weight of piglets was recorded on days 1, 15, and 35 in order to determine the average daily gain (ADG) on a pen-based diet, and the average daily feed intake (ADFI) was determined by checking the feed intake every day. The ADG and ADFI measurements were used to calculate the feed conversion ratio (FCR).

#### 2.3.2. Nutrient Digestibility

The piglets’ feed was supplemented with 0.5% chromium oxide, an indigestible marker, on the 28th day of the experiment and continued for approximately a week (until the trial’s conclusion) in order to gauge the nutrient digestibility. As soon as the feed was mixed, the corresponding feed samples from each treatment group were taken out using sterile plastic bags. Fresh fecal collection (2 pigs—1 barrow and 1 gilt/pen) was done by rectal palpation at the conclusion of each phase. The fecal samples obtained were taken to the laboratory and kept at −20 °C to avoid nutritional changes within 30 min. The fecal samples were dried for two days at 105 °C in a Daehan Scientific WOF-L800 convection oven drier prior to examination. After that, the samples were removed from the dryer and ground thoroughly using a Wilson mill so that they could go through a 1 mm screen sieve. The Association of Official Analytical Chemists International, AOAC [13] techniques were followed in the analysis of the apparent total tract digestibility (ATTD) of dry matter (DM), nitrogen (N), and gross energy (GE). In contrast, the organic matter was calculated using AOAC procedure 942.05 (AOAC International) to determine the ash content of the meal and fecal samples. To measure the chromium concentrations, a Shimadzu Ultraviolet (UV-1201) spectrophotometer was used. The Tector Kjeltec^TM^ 8400 (FOSS) analyzer (FOSS, Hillerød, Denmark) was used to measure the crude protein (N) content (Method 981.10 [14]). In contrast, GE was ascertained using a Parr 6400 oxygen bomb calorimeter (Parr Instrument, Moline, IL, USA).

##### Formula for ATTD

Nutrient digestibility (%) = [1 − (Nf × Cd)/(Nd × Cf) ] × 100, where Nf—number of nutrients in feces (% DM), Nd—number of nutrients in the diet (% DM), Cd—chromium content in the diet (% DM), and Cf—chromium content in feces (% DM).

#### 2.3.3. Fecal Score

Fresh samples were collected from the piglets during weeks 1, 2, and 5. On days 1, 15, and 35, feces were measured and recorded at 8:00 and 20:00 hat the pen source. The fecal score evaluation was done in accordance with the guidelines of Montagne [15]. The fecal score was determined for two pigs in each pen using the fecal scoring system described below: 1, firm, formed stools; 2, hard, dry pellets; 3, soft, moist stools that retain their shape; 4, unformed, soft stools that conform to the shape of the vessel; and 5, watery, pourable liquid.

#### 2.3.4. Blood Profile

On day 35 in all treatments, four piglets per treatment with average body weight for every replicate were randomly selected to collect 5 ml blood samples through venipuncture of the anterior vena cava using asterilized syringe. The samples were collected into disposable culture tubes. The blood samples were then brought to the lab (within 30 min) and centrifuged at 3000 rpm for 15 min at 4 °C and 1409× *g* using a centrifuge (model number MF-550, Hanil Science Industrial, Incheon, Republic of Korea) and were kept at −80 °C until analysis. These analyses included blood urea nitrogen (BUN), immune response (IgA, IgG), and insulin-like growth factor (IGF-1) at the two experimental phases (phase I and phase II). Following a careful transfer to 1.5 mL microtubes, the plasma was examined using commercially available enzyme-linked immunosorbent assay (ELISA) kits (ICL’s Pig IgA and IgG ELISA Kit). A microplate reader (VERSA Max; Molecular Device LLC, San Jose, CA, USA) set to 450 nm was used to measure the ideal density values of the standard and plasma samples. BUN was measured using a biochemical analyzer (Model 7020, Hitachi, Tokyo, Japan).

### 2.4. Statistical Analysis

All data were analyzed by ANOVA using the Duncan multiple range test method in SAS 9.1 Software (SAS Institute Inc., Cary, NC, USA). Using Duncan’s various comparison analyses, the significant difference was determined by comparing the means. The standard error of means (SEM) represented variation in data, and a value of *p* < 0.05 was considered statistically significant.

## 3. Results

### 3.1. Growth Performance

There was no significant difference in the growth performance of piglets fed a basal diet with dried mealworm, hydrolyzed mealworm, FPBM, and HFSP, as presented in Table 4. During the overall experimental period, TRT2 (dried mealworm) showed a higher ADG than the other groups, but the difference was not statistically significant.

### 3.2. Nutrient Digestibility

There was no significant difference in the apparent total tract digestibility (ATTD) of dry matter (DM), nitrogen (N), and apparent retention of energy (E), as shown in Table 5. TRT2 showed higher ATTD of DM and N and also higher N-retention than the other groups, but the difference was not statistically significant.

### 3.3. Fecal Score

The basal diet with dried mealworm, hydrolyzed mealworm, FPBM, and HFSP had no significant impact on the fecal score, as shown in Table 6. TRT2 was higher in D15 than the other groups, but the difference was not statistically significant, while TRT2 and TRT4 (hydrolyzed fish soluble) were higher in D35 than the other groups, but the difference was not statistically significant.

### 3.4. Blood Profile

There was a significant difference in the BUN concentration of the piglets fed a basal diet with dried mealworm, hydrolyzed mealworm, FPBM, and HFSP, as shown in Table 7. The blood urea nitrogen concentration was significantly different among dietary treatments. Significant differences were observed in BUN concentration but not in serum IGF-1, IgG, and IgA concentrations by the addition of dried mealworm, hydrolyzed mealworm, FPBM, and HFSP. FPBM decreased BUN concentration and had the lowest value compared to the other treatments, followed by hydrolyzed mealworm, which tended to be low but not significant, while dried mealworm and HFSP increased BUN concentration.

## 4. Discussion

In swine production, high-quality proteins are frequently used [10]. Pig age, cost, digestibility, and palatability all play a role in choosing the right protein sources [10]. Animal- or plant-based proteins have historically been utilized as feedstock. But according to Cho [16], conventional protein sources are no longer adequate to meet feed output growth in a sustainable manner. Due to weaning stress and the abrupt cessation of sow’s milk, piglets’ performance is severely compromised during the first few weeks of life, particularly 21 days after weaning [17]. To encourage feed intake post-weaning, highly palatable and nutrient-dense protein sources are often included in nursery diets [3]. Recently, complex diets incorporating highly palatable and easily digestible animal protein sources have been formulated to address this challenge [18]. Similarly to our study, Zier [19] found that using poultry by-product meal (PBM) as a substitute protein source for fish meal (FM) showed no significant difference in the overall performance of weaning pigs for the whole experimental period of 26 days. Cho [18] found that the addition of a 3% hydrolysate mealworm larvae diet did not show a significant effect on the growth performance of weaning pigs. Jones [3] observed no significant difference in ADG in nursery pigs fed 6% fish meal that has different levels of fish soluble protein (0.87%, 8.70%, 16.52%, or 24.35%) on nursery pigs. García-Rodriguez [20] observed that low-level supplementation (2%) of hydrolyzed fish protein (HFP) in pre-starter feeding did not have any negative effect on the growth performance of piglets after weaning. However, according to Xu [21], fermented feed supplementation improved the ADG and feed–gain ratio (F/G) of weaning piglets (ADG: weight mean difference = 20.869 g/day). Huang [22] found that 5% inclusion of a two-stage fermented feather meal soybean meal product (TSFP) increased ADG in nursery pigs. Young [23] observed a linear improvement in ADG. These dissimilarities may be due to the level of supplementation.

There was no significant effect on nutrient digestibility among the treatments. Ko [24] observed that using two diets, a combination of fish meal and dried mealworm diet (50% fish meal replaced with dried mealworm meal) and a dried mealworm diet (100% replacement fish meal with dried mealworm meal) showed no significant effect on the ATTD of DM and GE. Cho [18] found that the addition of 3% hydrolysate mealworm larvae diet did not show a significant effect on the ATTD of DM, N, and GE in weaning pigs. However, using a basal diet with 1% fish meal and dried mealworm (*Tenebrio molitor*) and a basal diet with 2% dried mealworm), Ao [25] found that the ATTD of nitrogen in the 1% dried mealworm diet was lower compared with the control diet. This difference from our study may be due to the level of supplementation and type of mixed feed supplementation.

There was no significant effect on fecal scores among the treatments. Wen [1] stated that different sources of protein (fish meal, 19% CP; fish meal, 23% CP; and other protein sources) did not have any difference on the fecal score of piglets that were weaned at 21 days of age. However, Zhe [26] observed a higher percentage of piglets with fecal scores of 2 or ≥2 when fed a diet containing spray-dried chicken plasma protein (5%).

In this study, there was a significant difference in BUN concentration. FPBM resulted in the lowest BUN concentration (6.3 mg/dL) compared to the other diets, followed by hydrolyzed mealworm (7.3 mg/dL), while BUL levels were higher with dried mealworm and HFSP (8.5 mg/dL). BUN can be used to measure the N utilization and excretion rate in pigs;decreased usage of dietary protein indicates high BUN concentrations [27]. Protein synthesis and nitrogen deposition can both rise when BUN levels decrease. The primary by-product of animal protein metabolism is BUN. The body’s urea content is influenced by liver function, tissue protein catabolism, and dietary protein consumption [28]. According to Yang [29], BUN levels can serve as indicators of nitrogen use and protein deposition, as well as reflect how animal protein is metabolized. Zhe [26] found that dietary supplementation with 5% spray-dried chicken plasma protein resulted in higher serum urea nitrogen levels compared to the control diet. Ko [24] observed that using two diets—a combination of fish meal and dried mealworm diet (50% fish meal replaced with dried mealworm meal) and a dried mealworm diet (100% replacement of fish meal with dried mealworm meal)—showed no significant effect on IgG concentration. In contrast to our study, Ao et al. [25] observed no significant difference in blood urea nitrogen levels when using a basal diet with 1% fish meal and dried mealworm (*Tenebrio molitor*) or a basal diet with 2% dried mealworm. Huang [22] found that inclusion of 5% two-stage fermented feather meal soybean meal product increased IgG concentration in nursery pigs. This indicates that consuming poultry by-products, which are also feed sources of animal protein, will have a positive impact on an animal’s health [8]. The dissimilarities of this study with other studies may be due to the percentage of feed additives used.

## 5. Conclusions

The inclusion of 10% fermented poultry by-products in the diets of weaning piglets is a beneficial protein source for improving blood profile by decreasing blood urea nitrogen, without any negative effect on growth performance, nutrient digestibility, fecal score, and other blood parameters. Also, the addition of dried mealworm, hydrolyzed mealworm, and hydrolyzed fish soluble protein in the same amount has no negative effect on the same parameters. Therefore, fermented poultry by-products can be used in diets as a beneficial alternative feed protein in weaning pig diets.

## Figures and Tables

**Table 1:** Composition of dried mealworm, hydrolyzed mealworm, FPBM, and HFSP ^1^.

Chemical Composition (%)	Dried Mealworm	Hydrolyzed Mealworm	FPBM	HFSP
Moisture	8.5	10.58	5.83	5.38
Crude protein	68.00	58.88	71.52	89.04
Crude fat	4.00	0.26	11.22	7.00
Crude ash	8.00	14.90	9.30	5.00
Ca	0.6	0.20	0.10	0.20
Total P	0.50	0.82	1.00	0.75
Amino acid				
Essential	36.76	30.05	31.44	19.24
Methionine	2.07	3.02	1.96	1.36
Cystine	0.87	0.75	0.67	0.15
Valine	4.78	3.02	3.89	1.80
Isoleucine	3.41	1.80	2.96	1.47
Leucine	7.07	3.77	6.09	2.90
Phenylalanine	3.74	2.07	3.44	1.76
Tyrosine	3.41	1.15	2.92	0.00
Histidine	1.49	0.92	2.89	1.52
Lysine	5.82	10.36	3.12	6.00
Threonine	4.10	3.19	3.50	2.28
Non-essential	45.99	33.17	47.81	49.04
Serine	6.18	3.19	3.80	3.89
Arginine	6.01	3.19	5.86	4.93
Glutamic acid	13.46	10.39	13.25	7.95
Aspartic acid	7.66	4.82	7.54	5.33
Proline	1.66	3.26	4.98	6.79
Glycine	6.12	4.48	6.61	13.98
Alanine	4.90	3.84	5.77	6.17

^1^ Abbreviations: FPBM—fermented poultry by-product mean; HFSP—hydrolyzed fish soluble protein.

**Table 2:** Composition of weaning pig diets (as fed-basis).

Item	Dietary Treatment (Phase 1)			
	T1	T2	T3	T4
Raw Material (%)				
Defatted mealworm larvae	10	-	-	-
Fermented poultry by-product meal	-	-	10	-
Hydrolysate fish meal	-	-	-	10
Hydrolysate of defatted mealworm larvae	-	10	-	-
Dextrin	10	10	10	10
Corn, 5 mm	28.4	18.16	24.2	27.7
Barley	4.64	12	12	12
Lactose	12	12	12	12
SBM, dehulled, 48	24.7	26	22.54	18.76
Whey powder	3	3	3	3
Soy oil	3.44	5.33	2.14	2.6
Limestone	1.04	1.13	1.21	1.15
MonocalPhosp	1.58	1.39	1.35	1.53
Salt	0.3	0.29	0.29	0.3
Methionine, 99%	0.03	-	0.12	0.08
Lysine, 78.4%	0.17	-	0.45	0.18
Choline, 50%	0.1	0.1	0.1	0.1
ZnO	0.1	0.1	0.1	0.1
Vit/Min premix ^1^	0.5	0.5	0.5	0.5
Total	100	100	100	100
Nutrient (Analysis)				
Moist	8.67	9.53	9.1	9.15
C:PRO	21.5	21.4	21.51	21.5
C:FAT	5.39	6.73	4.76	4.8
C:FIBER	1.83	2.08	2.09	2.02
C:ASH	5	5.12	5	4.88
CA	0.8	0.8	0.8	0.8
TP	0.65	0.65	0.65	0.65
LYS	1.35	1.48	1.35	1.35
MET	0.39	0.4	0.39	0.39
MES	3400	3400	3400	3400

^1^ Provided per kg diet: Fe, 100 mg as ferrous sulfate; Cu, 17 mg as copper sulfate; Mn, 17 mg as manganese oxide; Zn, 100 mg as zinc oxide; I, 0.5 mg as potassium iodide; and Se, 0.3 mg as sodium selenite. Provided per kilogram of diet: vitamin A, 10,800 IU; vitamin D3, 4000 IU; vitamin E, 40 IU; vitamin K3, 4 mg; vitamin B1, 6 mg; vitamin B2, 12 mg; vitamin B6, 6 mg; vitamin B12, 0.05 mg; biotin, 0.2 mg; folic acid, 2 mg; niacin, 50 mg; D-calcium pantothenate, 25 mg. C:PRO = crude protein, C:FAT = crude fat, C:FIBER = crude fiber, C:ASH = crude ash.

**Table 3:** Composition of weaning pig diets (as fed-basis).

Item	Dietary Treatment (Phase 2)			
	T1	T2	T3	T4
Raw Material (%)				
Defatted mealworm larvae	10	-	-	-
Fermented poultry by-product meal	-	-	10	-
Hydrolysate Fish meal	-	-	-	10
Hydrolysate of defatted mealworm larvae	-	20	-	-
Dextrin	10	-	10	10
Corn, 5 mm	25	20.7	26.9	27.2
Barley	30	30	30	30
Lactose	4	4	4	4
SBM, dehulled, 48	14.2	17.41	13.38	12.96
Soy oil	3.07	4.76	1.7	2.19
Limestone	0.95	1.04	1.12	1.05
MonocalPhosp	1.45	1.24	1.21	1.34
Salt	0.25	0.15	0.3	0.3
Methionine, 99%	0.04	-	0.11	0.06
Lysine, 78.4%	0.34	-	0.58	0.2
Choline, 50%	0.1	0.1	0.1	0.1
ZnO	0.1	0.1	0.1	0.1
Vit/Min premix ^1^	0.5	0.5	0.5	0.5
Total	100	100	100	100
Nutrient (Analysis)				
Moist	9.37	10.5	10.04	10.02
C:PRO	18.5	18.5	18.85	20.09
C:FAT	4.99	6.24	4.31	4.42
C:FIBER	2.83	2.86	2.85	2.83
C:ASH	4.5	4.5	4.5	4.5
CA	0.7	0.7	0.7	0.7
TP	0.6	0.6	0.6	0.6
LYS	1.23	1.27	1.23	1.23
MET	0.36	0.37	0.36	0.36
MES	3350	3350	3350	3350

^1^ Provided per kg diet: Fe, 100 mg as ferrous sulfate; Cu, 17 mg as copper sulfate; Mn, 17 mg as manganese oxide; Zn, 100 mg as zinc oxide; I, 0.5 mg as potassium iodide; and Se, 0.3 mg as sodium selenite. Provided per kilogram of diet: vitamin A, 10,800 IU; vitamin D3, 4000 IU; vitamin E, 40 IU; vitamin K3, 4 mg; vitamin B1, 6 mg; vitamin B2, 12 mg; vitamin B6, 6 mg; vitamin B12, 0.05 mg; biotin, 0.2 mg; folic acid, 2 mg; niacin, 50 mg; D-calcium pantothenate, 25 mg. C:PRO = crude protein, C:FAT = crude fat, C:FIBER = crude fiber, C:ASH = crude ash.

**Table 4:** Effect of different protein sources on growth performance of weaning pigs ^1^.

Item	TRT1	TRT2	TRT3	TRT4	SEM ^2^	*p*-Value
Body weight, kg						
Initial	7.94	7.94	7.96	7.95	0.01	0.209
D15	12.49	12.95	13.12	12.84	0.15	0.079
D35	24.65	25.63	25.40	24.78	0.27	0.095
D15						
ADG, g	304	333	344	325	10	0.091
ADFI, g	399	428	440	421	17	0.215
FCR	1.312	1.284	1.279	1.295	0.015	0.183
D35						
ADG, g	608	634	614	598	14	0.154
ADFI, g	924	946	922	904	20	0.274
FCR	1.520	1.492	1.5	1.512	0.032	0.371
Overall						
ADG, g	478	505	499	481	8	0.123
ADFI, g	699	724	715	697	15	0.315
FCR	1.464	1.433	1.435	1.449	0.025	0.237

^1^ Abbreviations: TRT1, dried mealworm; TRT2, hydrolyzed mealworm; TRT3, fermented poultry by-product meal; TRT4, hydrolyzed fish soluble protein. ^2^ Standard error of means.

**Table 5:** Effect of different protein sources on nutrient digestibility of weaning pigs ^1^.

Items, %	TRT1	TRT2	TRT3	TRT4	SEM ^2^	*p*-Value
Finish						
Dry matter	79.04	80.28	79.81	78.77	1.38	0.444
Nitrogen	77.29	78.35	77.82	76.67	1.26	0.239
N-retention	3.19	3.22	3.12	3.05	0.10	0.158

^1^ Abbreviations: TRT1, dried mealworm; TRT2, hydrolyzed mealworm; TRT3, fermented poultry by-product meal; TRT4, hydrolyzed fish soluble protein. ^2^ Standard error of mean.

**Table 6:** Effect of different protein sources on fecal scores for weaning pigs ^1^.

Item	TRT1	TRT2	TRT3	TRT4	SEM ^2^	*p*-Value
Fecal score ^3^						
Initial	3.50	3.52	3.54	3.51	0.02	0.128
D15	3.44	3.46	3.44	3.44	0.03	0.561
D35	3.21	3.22	3.16	3.22	0.04	0.177

^1^ Abbreviations: TRT1, dried mealworm; TRT2, hydrolyzed mealworm; TRT3, fermented poultry by-product mean; TRT4, hydrolyzed fish soluble protein. D15 = day 15, D35 = day 35. ^2^ Standard error of mean. ^3^ Score = 1 = hard, dry pellets in a small, hard mass; 2 = hard, formed stool that remains firm and soft; 3 = soft, formed, and moist stool that retains its shape; 4 = soft, unformed stool that assumes the shape of the container; 5 = watery, liquid stool that can be poured.

**Table 7:** Effect of different protein sources on the blood profile of weaning pigs ^1^.

Item	TRT1	TRT2	TRT3	TRT4	SEM ^2^	*p*-Value
Finish ^3^						
BUN, mg/dL	8.5 ^a^	7.3 ^ab^	6.3 ^b^	8.5 ^a^	0.8	0.019
IGF-1, ng/dL	136.0	139.5	122.2	147.3	13.1	0.240
IgG, mg/dL	222.8	233.5	250.8	265.3	20.0	0.111
IgA, mg/dL	191.5	188.0	193.0	192.8	12.7	0.803

^1^ Abbreviations: TRT1, dried mealworm; TRT2, hydrolyzed mealworm; TRT3, fermented poultry by-product meal; TRT4, hydrolyzed fish soluble protein. ^2^ Standard error of mean. ^3^ Means in the same row with different superscripts differ significantly (*p* < 0.05).

## Data Availability

Data are available on request due to privacy.
